# Deep Learning-Based Tracking of Neurovascular Features Toward Semi-Automated Ultrasound-Guided Peripheral Nerve Blocks by Non-Specialists

**DOI:** 10.3390/bioengineering13050556

**Published:** 2026-05-15

**Authors:** Lars A. Gjesteby, Alec Carruthers, Joshua Werblin, Nancy DeLosa, Carlos Bedolla, Mateusz Wolak, Benjamin W. Roop, Elizabeth Slavkovsky, Sofia I. Hernandez Torres, Krysta-Lynn Amezcua, Eric J. Snider, Samuel B. Kesner, Brian A. Telfer, Brian J. Kirkwood, Laura J. Brattain

**Affiliations:** 1 MIT Lincoln Laboratory, Lexington, MA 02421, USA; lars.gjesteby@ll.mit.edu (L.A.G.); alec.carruthers@ll.mit.edu (A.C.); mateusz.wolak@ll.mit.edu (M.W.); benjamin.roop@ll.mit.edu (B.W.R.); elizabeth.slavkovsky@ll.mit.edu (E.S.); samuel.kesner@ll.mit.edu (S.B.K.); laura.brattain@ucf.edu (L.J.B.); 2 Organ Support & Automation Technology Research Department, United States Army Institute of Surgical Research, Fort Sam Houston, San Antonio, TX 78234, USAbrian.j.kirkwood.mil@health.mil (B.J.K.)

**Keywords:** peripheral nerve block, ultrasound, artificial intelligence, deep learning, femoral nerve block

## Abstract

Peripheral nerve blocks can effectively reduce the use of general anesthesia and opioids in situations where robust pain management is critical, such as severe extremity trauma and hip, femur, and knee surgeries. Despite these benefits, nerve blocks are underutilized due to the high skill required to accurately insert a needle and safely deliver local anesthetic. To overcome this challenge, ultrasound image guidance enabled by artificial intelligence (AI) offers a semi-automated solution for regional anesthesia delivery by non-specialists. As a first step towards realizing an integrated platform for AI-guided nerve blocks, the main objective of this study is to develop and characterize deep learning algorithms to interpret anatomical landmarks on ultrasound images in real time and identify aimpoints for needle placement. Our AI system was trained on over 55,000 images from 20 porcine models and demonstrated an average area under the precision–recall curve of 0.92 (SD = 0.03) for in vivo landmark detection in the femoral nerve region. In prospective live animal testing, aimpoint identification had a 98.3% success rate with an average time of 40.5 s (SD = 33.5). Future work will focus on integrated testing with handheld robotics towards a more accessible method for delivering regional anesthesia in settings from point of injury to medical transport to hospitals.

## 1. Introduction

Regional anesthesia targets specific structures with injections of local anesthetics or analgesics, and regional anesthesia techniques like peripheral nerve blocks are increasingly used for localized pain management [1]. Regional anesthesia is less expensive, not addictive, more targeted, and has fewer complications than other pain management techniques, such as general anesthesia and opioid administration [1,2,3,4,5]. Ultrasound guidance improves the administration of local anesthesia, with better efficacy and safety over blind techniques such as palpation or nerve stimulation [6]. Additionally, ultrasound guidance leads to less local anesthesia by volume and a shorter time to achieve the block [7]. These benefits are particularly valuable in settings where medical equipment and trained staff are limited.

Pain management is critical in challenging environments, such as battlefield care for wounded military personnel and emergency response scenarios with limited medical resources. Nerve blocks provide localized pain relief, allowing patients to remain alert and communicative while avoiding the risks of opioid use. Portable ultrasound devices have improved the feasibility of nerve blocks in austere settings, but their effectiveness depends on personnel trained in both ultrasound and regional anesthesia techniques, which remains a significant barrier to widespread adoption. Addressing this training gap is essential to fully realize the potential of nerve block methods in resource-constrained environments [8,9]. Additionally, this training gap even extends to well-resourced hospitals [10]. With 790,000 knee replacements and 544,000 hip replacements completed annually in the U.S. [11], enabling medical staff with less specialized training to perform femoral peripheral nerve blocks could benefit patients who want or need alternative pain management options.

While ultrasound assistance for regional anesthesia has many advantages, interpreting the images requires personnel to have extensive training and expertise. Ultrasound alone does not provide guidance to clinicians for identifying anatomical landmarks or determining an aimpoint safely [12]. Previous studies have shown that it can be challenging to locate a specific nerve initially and keep it in view during the nerve block [13]. Due to variations in the angle of needle insertions, the needle, particularly the tip, can disappear from the ultrasound field of view [13]. Further, site-specific peripheral nerve blocks have several safety and efficacy challenges. The anesthetic must be injected close to the nerve but must not injure the nerve. If the aimpoint near the nerve is not correctly identified, adverse effects include bleeding, peripheral nerve injury, local anesthetic toxicity due to misplaced injections or accidental anesthetic injection into the nerve, and potential fatalities due to injecting anesthetics into blood vessels [14,15]. One study of 2735 encounters showed that adverse events are rare, with one major complication of local anesthetic toxicity and nine minor complications including hoarseness, breathing difficulty, hypotension, numbness and weakness, totaling 0.4% of cases [15]. However, pain relief outcomes vary significantly. In one cohort study, 70.8% of patients experienced 51–100% pain relief, but 11.3% required additional analgesia or general anesthesia due to minimal improvements in pain scores. Operator experience and training may have had a role in the inaccurate targeting of local anesthesia [15].

Given these challenges, consistent and comprehensive training are essential. Unfortunately, current education for regional anesthesia in the United States varies by program. Because not all anesthesiologists have consistent training on regional nerve blocks, this can lead to inequities for patient access for specific blocks. Often an anesthesiologist specializes in a particular set of blocks, leading to different access availability for different patients, even at the same institution [10].

To address the aforementioned gaps, artificial intelligence (AI) has emerged as an effective tool to assist clinicians in identifying neurovascular structures and guide robotic needle placement [16,17,18]. However, existing methods fall short in being able to both automate the calculation of a needle aimpoint and provide real-time guidance in portable form factor for prehospital and field settings. Our work presents the initial algorithm development of the Artificial Intelligence Guided Ultrasound Interventional Device for Nerve Block (AI-GUIDE Nerve Block), a handheld ultrasound-based prototype designed to identify nerve region structures and target an aimpoint for local anesthesia delivery. The platform was derived from the AI-GUIDE vascular access device, which integrated vessel detection algorithms to automate needle insertion into deep blood vessels with custom handheld robotics [19]. The core AI methods used in AI-GUIDE are based on the YOLO family of models, a widely used class of deep learning models for computer vision tasks like object detection and segmentation. This class of model has been deployed in diverse applications including medical image analysis, transportation, and security monitoring [20]. Despite similarities between the vascular access and nerve block devices, nerve block procedures pose distinct challenges compared to vascular access. For example, the nerves themselves present with more variation in appearance on ultrasound as compared to veins and arteries [13], which makes the image processing more difficult.

**Figure 1:**
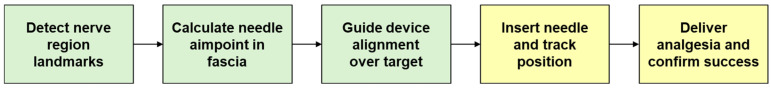
Operational overview for AI-GUIDE Nerve Block. This paper discusses development of items in green, including interpreting the nerve region landmarks in ultrasound, locating an aimpoint for needle insertion into the fascia, and providing guidance to the operator. Future research efforts (in yellow) include inserting, tracking, and adjusting the needle position, as well as injecting and confirming analgesia location and efficacy.

Our results include algorithms to interpret ultrasound images, detect nerve region landmarks, and calculate an aimpoint for needle insertion into the nearby fascia (Figure 1, in green). Porcine testing of these capabilities showed that the initial workflow steps can be efficiently completed by users with limited medical expertise. Testing was conducted under both normovolemic and hypovolemic conditions to evaluate the developed AI model performance in hypothetical emergency scenarios, which may include hemorrhagic shock. The femoral nerve is the most studied block within the relatively new field of ultrasound-guided regional anesthesia, so initial studies focused on that specific procedure [14].

We recognize that the image interpretation for determining a needle aimpoint is just one step in a complex nerve block workflow. Thus, future development efforts will expand on this foundation by incorporating advanced capabilities, including insertion, tracking, and adjustment of the needle for reliable placement, as well as injecting analgesia and confirming its location and efficacy (Figure 1, in yellow). These enhancements will be critical for improving the device’s functionality and ensuring safe and effective administration of regional anesthesia in diverse care settings.

The goal with the AI-GUIDE Nerve Block device is to make effective nerve block techniques possible for many more health professionals in more environments, improving outcomes in pain management across a variety of clinical settings. The work presented here on developing AI models is a first significant step towards this long-term goal.

## 2. Materials and Methods

### 2.1. Data Description

Ultrasound scans of swine were collected by sonographers at the United States Army Institute of Surgical Research (USAISR) and at Massachusetts General Hospital (MGH) using a Terason uSmart 3200T Plus ultrasound device (Terason, Burlington, MA, USA). All data collections at each respective site were approved by the Institutional Animal Care and Use Committee (IACUC). The use of a swine model, which is physiologically similar to humans, supports the potential for translating this technology to clinical use. A total of 55,455 B-mode ultrasound images of the femoral region from the left and right legs of 20 swine, including 17 in vivo and 3 ex vivo, were collected for algorithm development. The images from MGH were collected on female Yorkshire swine weighing 50–70 kg. The images from USAISR we collected on healthy female Yorkshire crossbred swine (Sus scrofa domestica), approximately 3–4 months old, weighing 35–50 kg, and free of primary pathogens. Anesthesiologists oversaw scanning procedures to collect data centered around ideal anatomical sites for a nerve block, and subsequent scans started and ended at this location, including bidirectional rotation, compression, proximal to distal, medial to lateral, lateral to medial, and 45-degree angulation sweeps (proximal to distal). For all images in the dataset, bounding boxes were drawn with domain expert input around relevant anatomical features for performing the nerve block, including the femoral artery (FA), femoral vein (FV), and fascia iliaca (FI), which is a fibrous layer around the femoral nerve. Segmentation masks of the FI were drawn on 53,877 images in the dataset. The aimpoint was calculated as the centroid of the FI segmentation mask.

Additionally, 13,668 B-mode ultrasound images were collected at USAISR from the sciatic nerve (SN) region on the left and right legs of four swine. Bounding boxes were drawn around the SN, a hyperechoic region between the adductor magnus and biceps femoris muscles [21]. No aimpoint or needle tracking was labeled or defined for the SN; however, future work plans to extend this approach to defining segmentation masks for the SN. Table 1 summarizes the datasets used for algorithm training and validation.

### 2.2. Ex Vivo Swine Model

An ex vivo model of the lower half of a swine was used for a portion of the data collection and initial device testing. This same re-perfused model has been described previously for vascular access procedures and testing automated vascular access devices [22,23]. Catheters were secured at the artery and vein proximal and distal to the inguinal crease area where imaging will occur. At the distal site, the loop was connected via an arterio-venous shunt allowing return flow from the arterial side through the venous side. The loop circulated water via a MasterFlex pump (Avantor, Radnor, PA, USA), with a pressure sensor connected to a patient monitor to ensure physiological pressures. The pump flow rate was adjusted to maintain hypotensive model conditions. Any branching vessels were ligated to limit water leakage and edema build-up. The tissue around the inguinal crease was kept intact to maintain ultrasound image quality.

### 2.3. Algorithm Training Details

This study evaluated object detection and segmentation algorithms for femoral landmarks trained on both in vivo and ex vivo porcine data. The detection model was developed for the FA, FV, and FI, while the segmentation model was developed only for the FI for aimpoint identification. In a standard ultrasound-guided femoral block, a clinician identifies the FI and an aimpoint in the FI, as the local anesthesia is injected to surround the nerve. The needle must not puncture the nerve itself, as that can lead to permanent nerve damage. The FI is also more visible in the ultrasound image than the femoral nerve.

A training–validation–testing split of 70:10:20 was used to train and evaluate the performance of a You-Only-Look-Once version 8 nano (YOLOv8n) object detection model in PyTorch 2.8 for femoral landmark detection [24,25,26,27]. YOLOv8n was chosen for its state-of-the-art balance of real-time capability and accuracy at the time of this study. The percentage splits were performed at a subject / swine level to prevent training data from contaminating the validation or test sets. With data from only three ex vivo swine, one was used in each split to enable model comparison between in vivo and ex vivo swine. A hyperparameter sweep was performed across the batch size, learning rate, optimizer, weight decay, image augmentation settings (reflection about the vertical axis, translation, mosaicking, rotation, color jitter), optimizer, and image size. Image resizing was performed with padding of the smaller dimension to preserve the aspect ratio of the original ultrasound image. The learning rate was linearly increased across the first five epochs, reaching a maximum learning rate shown in Table 2, then decayed following a cosine annealing schedule. The final settings for these parameters, shown in bold in Table 2, were then used in a five-fold cross validation training of the model. Each fold was set to train for 100 epochs on a single NVIDIA H100 GPU, but training was halted if performance on the validation set did not improve for 10 epochs.

A YOLOv8n segmentation model was trained for FI segmentation using a similar procedure as outlined for femoral detection training, with a key difference of increasing the image size from 320 × 320 to 640 × 640. We observed worse precision–recall performance on the validation sets when the image resolution was coarser than 640 × 640.

The optimal hyperparameters found for the femoral detection model were also used to train a preliminary sciatic nerve detection model. With the smaller dataset from four total swine, four-fold cross validation was performed such that each swine served as the test set for one given iteration with the remaining data used for training and validation.

### 2.4. Evaluation Metrics

To evaluate the detection models, precision–recall curves were generated at an intersection over union (IOU) threshold of 0.5 between the model output bounding boxes and the ground truth bounding boxes for each class. The area under the curve was calculated as a measure of algorithm performance for each class and all classes together. To evaluate the segmentation model, the Dice coefficient was calculated as a measure of overlap between the model segmentation mask output and the ground truth mask of the fascia iliaca, with 1.0 representing maximum overlap.

### 2.5. Software Design and Workflow

We developed a custom C++ software application (app) on the Terason uSmart 3200T Plus to run image processing and deep learning algorithms in real time (30 Hz) on the ultrasound imaging feed. The Terason software development kit (SDK) version 5.12 was used as a starting point and implemented on a Windows 10 tablet with a NVIDIA Quadro P3000 1280 CUDA core graphics processing unit (GPU). The SDK allows independent software apps to communicate with the Terason ultrasound app, uSmart 3200T. The SDK enables passing of images from the uSmart 3200T app to our custom AI-GUIDE Nerve Block app and allows setting and receiving imaging parameters such as depth and gain. The SDK uses COM Automation, Active Object Registration, and the Windows MFC Library. The AI-GUIDE Nerve Block app was designed to determine where anatomical landmarks (including arteries, veins, and fascia) were located in the ultrasound image supplied via the SDK. Using this information, the app guides the device operator to position the device over the correct aimpoint.

Multi-threading was employed to integrate the detection and segmentation algorithms into the AI-GUIDE Nerve Block app for real-time image interpretation and guidance. NVIDIA TensorRT and Open Neural Network Exchange (ONNX) were used to optimize the trained PyTorch models to deliver low-latency and high-throughput on the NVIDIA GPU. The detection model had a runtime speed of 20 ms per 320 × 320 frame when deployed as an ONNX model on the device with no further optimizations. The higher 640 × 640 resolution of the segmentation model was addressed by implementing a TensorRT engine and CUDA C++ pipeline to run inference at 10 ms per frame. For comparison, the ONNX version of the segmentation model ran inference at 100 ms per frame. The deployed segmentation model had 3.4 million parameters, occupying a total memory of 14 MB. The total time for the algorithm chain to process a frame ranged between 30–50 ms. This workflow can support real-time image overlay of AI outputs for ultrasound imaging rates of 20–30 Hz. The app main thread sends ultrasound images to the algorithm thread, which then returns the generated array of bounding boxes and segmentation masks detected in the images. Each of the detections returned is reviewed to make sure it is valid to be passed back to the main thread. Only the object with the highest probability in each class is kept. For example, if the FA and FV class detections overlap by greater than 75%, then the higher probability detection is kept and the next highest probability detection of the other vessel is used if it is available and below the overlap threshold. Temporal information is also used by the app to determine an estimated location of landmarks based on detections from the five most recent frames if no detections are found on the current frame. This algorithm was based on empirical analysis of precision–recall metrics to determine an optimal number of frames for detections to persist.

**Figure 2:**
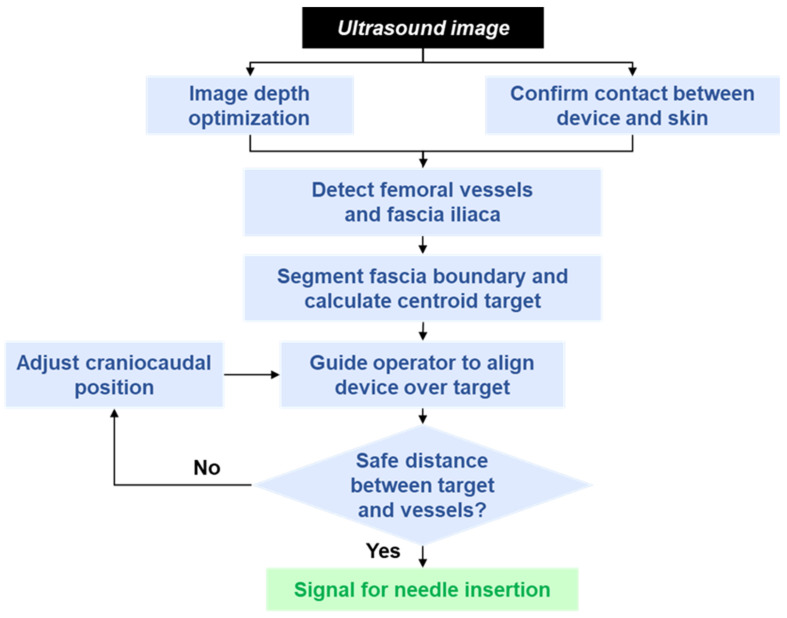
Image processing workflow of the AI-GUIDE Nerve Block software application. The algorithm modules include deep learning-based detection/segmentation and formulaic calculation of relative landmark positions. After receiving an ultrasound image, the app confirms contact and optimizes the image depth. Next, it detects the femoral vessels and fascia iliaca, then segments the fascia boundary to determine a needle aimpoint. It then gives the operator instructions to move the device to the target and adjust the position to ensure safe alignment. Black indicates input. Blue indicates an algorithm module. Green indicates outputv.

As shown in the flowchart in Figure 2, the first step in the image processing workflow is to confirm contact between the device and skin and to optimize the depth of the ultrasound image to ensure good visualization of the landmarks using our previously developed algorithms [28]. In general, image quality can suffer when deeper than necessary imaging depths are used, so choosing an appropriate depth for the subject-specific anatomy helps maximize device utility.

The initial depth for calibration was set to 6 cm for broad visibility, and the femoral vessels were used as the landmarks for which to optimize the depth. Once one or more vessels are detected, the depth calibration process calculates a buffer of approximately 1 cm relative to the image bottom using the deepest vessel bounding box. Adjustments are made by rounding up to the nearest integer. Once the depth is optimally set, the detection and segmentation algorithms calculate the needle aimpoint, and then provide guidance to the operator to align the device over the aimpoint. Once that position is found, a final algorithm checks to ensure the aimpoint in the FI is sufficiently far away from the edge of the FA for safe needle insertion (1 mm by default). If the needle aimpoint is too close, the operator is instructed to reposition the device cranial or caudal at their discretion until the safety margin is met [29,30]. When a safe distance is confirmed, the device displays a green box.

### 2.6. AI-GUIDE Device Integration

**Figure 3:**
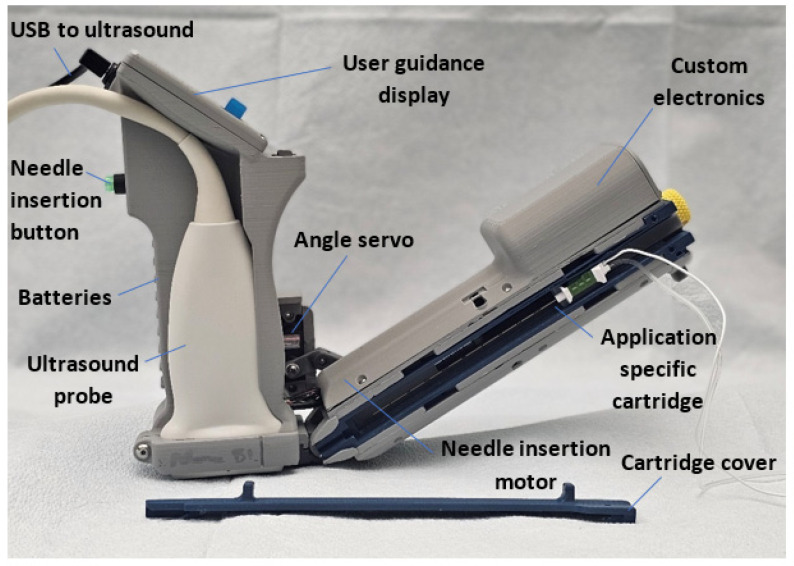
The full prototype AI-GUIDE-Nerve Block device includes two connected arms, including one arm that holds an ultrasound probe, user guidance display, and a needle insertion button, and the other holds a cartridge for a needle to be inserted with a motor.

AI-GUIDE is an ultrasound-based percutaneous intervention platform technology that was originally prototyped for vascular access as previously described in [19]. The device was adapted for nerve block applications as shown in Figure 3, and is still under development. It combines a housing for a commercial ultrasound probe, a display screen for user guidance, an arm that contains a needle cartridge and custom electronics to control the needle angle, and a button designed to insert the needle when the algorithm locates a suitable aimpoint. The device interfaces with the Terason uSmart 3200T Plus commercial ultrasound system running on a Windows 10 tablet. All prospective testing in this study was conducted using the handheld device running the AI-GUIDE Nerve Block software.

### 2.7. Prospective Testing Methods

Prospective testing of aimpoint identification and guidance was completed through in vivo and ex vivo porcine experiments. The ex vivo model was used for four rounds of testing (N = 11 ex vivo swine) with the device, making software updates in between testing rounds based on feedback from the users. Testing rounds allowed each user to scan using the device for up to 10 min (600 s) or until an aimpoint was identified and the device was aligned over it. If the aimpoint alignment was achieved, the time needed to do so was recorded. Otherwise, 10 min was recorded as the trial time. During the first two rounds of testing, the device was operated by several users with different levels of experience (engineers, clinicians, and animal technicians) to obtain feedback from various perspectives. During the last two rounds of testing, we downselected to the top two performing users, based on quicker aimpoint targeting rates, to conduct a more focused assessment of device performance prior to moving into in vivo porcine studies. The number of attempts for each ex vivo tissue model was limited by the image quality and tissue deterioration due to edema buildup.

For the second round of testing, the device was tested in live swine models from two approved animal protocols during June through August 2024 at USAISR. Research was conducted in compliance with the Animal Welfare Act, the Animal Welfare regulations, and the principles of the Guide for the Care and Use for Laboratory Animals [31,32]. The Institutional Animal Care and Use Committee at the United States Army Institute of Surgical Research approved all research conducted in this study. The facility where this research was conducted is fully accredited by the AAALAC International. Live animal subjects were maintained under a surgical plane of anesthesia and analgesia throughout the studies. For one of protocols, the swine were modeling an acute kidney injury and then were monitored for 24 h followed by humane euthanasia. The device testing occurred right before euthanasia, when the subject was normotensive. The second animal protocol was evaluating automated resuscitation controller performance during hemorrhagic shock in tandem hemorrhage–resuscitation events. This allowed for evaluating performance of the AI-GUIDE Nerve Block device in both normotensive and hypotensive conditions, before and after the second hemorrhage, respectively. The proof-of-concept testing of the device was a secondary objective in the swine study. After anesthesia, splenectomy, controlled hemorrhage, and resuscitation, the device was used to scan the inguinal crease of the swine. This test was conducted by a single user and timed from when scanning began to when a needle aimpoint was identified. For hypotensive testing, the process was repeated after the swine underwent a second hemorrhage to a mean arterial pressure of 35 mmHg. The maximum trial time was capped at 3 min (180 s). If the device was not successfully aligned over a calculated aimpoint within this time limit, the trial was stopped and a time of 180 s was recorded.

The device was tested in vivo in a total of eight swine on the right and left femoral nerves, with multiple tests for each, totaling 60 trials. Results on time-to-aimpoint identification were collected from animals under both normovolemic and hypovolemic conditions, as both conditions are likely in severe trauma scenarios. During testing, the operator could choose the aimpoint calculation method depending on the ability of the device to segment the FI. If the algorithm was not segmenting the FI reliably, the device operator could switch the system to calculate the needle aimpoint from the bounding box detection instead.

## 3. Results

### 3.1. Femoral Landmark Detection

**Figure 4:**
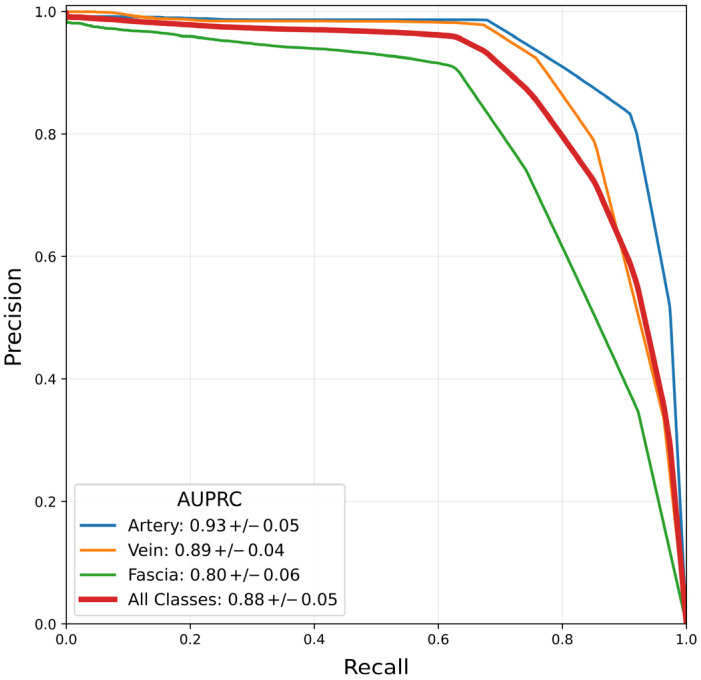
Precision–recall curves of the femoral nerve region landmark detection model tested on the full test set including both in vivo and ex vivo data. The model achieved area under the precision–recall curve values of 0.93 for the artery, 0.89 for the vein, and 0.80 for the fascia, with an overall precision–recall of 0.88 across all classes.

Figure 4 shows the precision–recall (PR) curve from five-fold cross-validation of the femoral landmark detection algorithm on the combined test set of in vivo and ex vivo images. The average area under the precision–recall curve (AUPRC) was 0.88 across all classes, 0.93 for the FA, 0.89 for the FV, and 0.80 for the FI. The FI tends to deform more than the FA and FV, making consistent identification of its anatomical features more challenging, leading to lower model performance [13,33].

**Figure 5:**
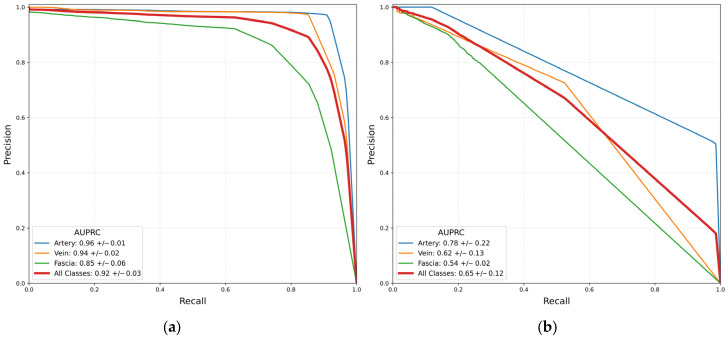
Precision–recall curves of the femoral landmark detection model tested on subsets of the test set: in vivo data (**a**) and ex vivo data (**b**). The overall area under the precision–recall curve was 0.92 for in vivo and 0.65 for ex vivo.

The model’s all-class average AUPRC was 0.92 for in vivo test data only, while the average AUPRC for ex vivo test data was 0.65, as shown in Figure 5. This marked difference in performance between in vivo and ex vivo test data is likely a result of the training dataset being heavily imbalanced in favor of in vivo data. Additionally, the ex vivo tissue degrades post-mortem as time passes, so image quality tends to be less clear.

### 3.2. Fascia Iliaca Segmentation

**Figure 6:**
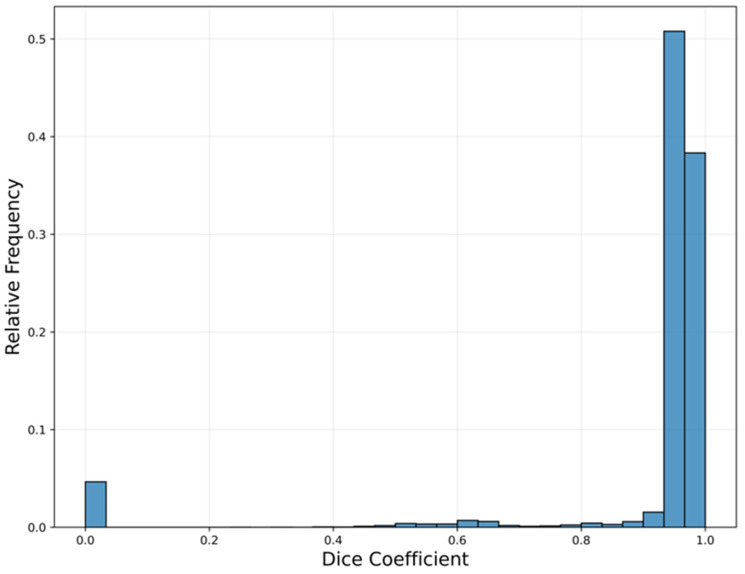
Relative frequency of Dice similarity coefficients between the fascia iliaca segmentation model output and the ground truth masks on the test set. The ideal Dice coefficient is 1.0. Results show that 91% of the model’s output segmentation masks were greater than 0.90, with false negatives at 0.0 primarily due to the model’s poor performance on segmenting the FI when it is compressed.

The FI segmentation model achieved mean and median Dice similarity coefficients of 0.91 and 0.96, respectively, across all test set images relative to ground truth FI masks. Figure 6 presents the distribution of Dice coefficients, indicating that 91% of the model’s output segmentation masks matched with the ground truth mask at greater than 0.90. The false negatives observed at the far left of Figure 6 are largely a result of the model’s poor performance on the FI when it is compressed, distorting the image, which reduces the area of the FI. Overall, however, the high level of performance achieved by this model enables reliable estimation of an aimpoint for needle insertion into the FI adjacent to the femoral nerve.

### 3.3. Prospective Testing of Aimpoint Identification

**Figure 7:**
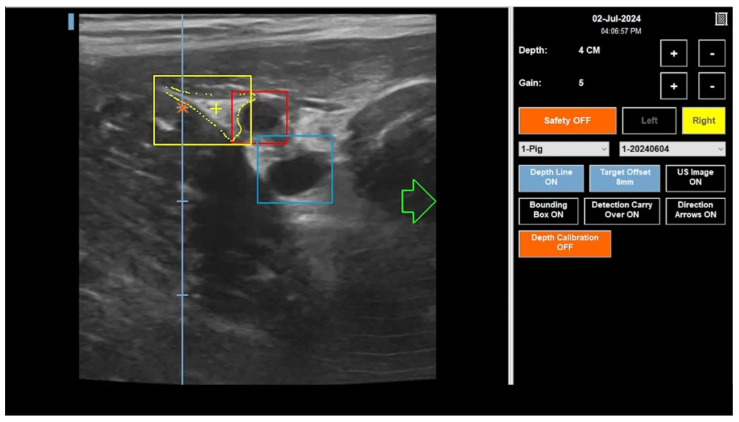
Screenshot of the custom AI-GUIDE Nerve Block software application displaying algorithm outputs—fascia iliaca detection (yellow box) and segmentation (yellow outline), femoral artery detection (red), and femoral vein detection (blue)—on the ultrasound image feed in real time in transverse view of the porcine anatomy. The orange “x” indicates the location where the hypothetical needle tip will be inserted to, and the yellow “+” indicates the calculated target aimpoint within the fascia iliaca to align with the “x”. The green arrow indicates the direction in which operator should move the device to align the target aimpoint with the needle path. The light blue vertical line indicates the depth of the image, with each tick mark representing 1 cm. The small blue rectangle in the upper left corner of the image corresponds to the mark on side of the ultrasound probe, which indicates the orientation of the image. The full video demonstration is included in Video S1.

The femoral algorithms were integrated into a custom AI-GUIDE Nerve Block software application to interpret ultrasound images in real time and display AI model outputs for user guidance (Figure 7). A video demonstration is included in Video S1. The operational workflow of the device emulates the clinical workflow of a nerve block. The operator first places the device in the target region on the patient, then scans the region by moving the device mediolaterally as instructed by the LCD display on the device. When the AI algorithm detects the target landmarks, the app guides the operator to align the device over the aimpoint for hypothetical needle insertion.

A total of 11 ex vivo porcine models were used during the first phase of prospective testing. Eight operators of varying experience levels, drawn from a multidisciplinary group of engineers, animal technicians, and clinicians, utilized the device to reach an aimpoint location determined by the AI models. A summary of ex vivo testing is shown in Table 3. The time to identify needle aimpoint decreased as device updates were implemented across four rounds. Initial rounds of testing were more exploratory, resulting in longer scanning times and fewer aimpoint identification successes. As more training data were added to the AI algorithms after each round, performance of the system improved. During Round 1 of testing, a total of nine aimpoint identification attempts were made with only two successes. The third ex vivo model in Round 1 had poor tissue quality, thus no attempts were made. Round 2 had 28 successful aimpoint identifications out of 38 attempts (73.7%), Round 3 had 65 successes out of 67 attempts (97.0%), and Round 4 had 43 successes out of 46 attempts (93.5%). The high success rates of needle aimpoint identification in Rounds 3 and 4 were accompanied by average attempt times of 47.7 ± 46.2 s and 96.1 ± 100.4 s, respectively. Round 4 of testing included two ex vivo models, one of which had poor tissue quality resulting in longer scanning times per attempt. The results of the final two rounds of testing showed that the system could consistently identify needle aimpoints and was ready for in vivo testing.

**Figure 8:**
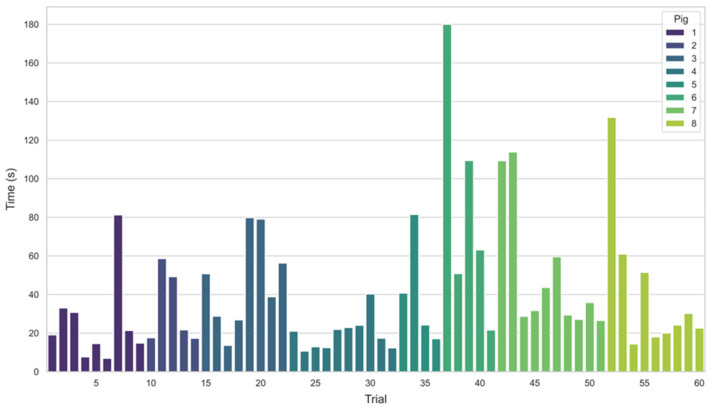
Times for the image processing algorithms to guide the operator to a suitable target aimpoint for hypothetical needle insertion. The average time was 40.5 s across 60 trials on eight in vivo porcine models.

For in vivo prospective testing, a total of 60 trials were attempted by two operators across eight live swine. Successful aimpoint identification was achieved in 59 trials (98.3%). Figure 8 presents the time-to-aimpoint identification that was recorded for each trial. This data excludes four trials in which the software application froze intermittently, one trial where timing data was not collected, and one trial where the animal died mid-trial. The maximum trial time was capped at 3 min (180 s). If the device was not successfully aligned over a calculated aimpoint within this time limit, the trial was stopped and a time of 180 s was recorded. The maximum time was reached in only one of the 60 trials.

The time to identify the target location in the fascial space varied across the eight test swine, with an average time of 40.5 s and a standard deviation of 33.5 s. Under normovolemic conditions, the average time to identification was 45.7 s (SD = 37.9) across 35 trials. Under hypovolemic conditions, the average time was 33.2 s (SD = 27.0) across 25 trials, indicating that the algorithm is robust to variations in blood volume and shock conditions.

While most of the aimpoints were identified based on the centroid calculated by the FI segmentation model, the user had the option to switch the aimpoint calculation to the detection model bounding box if the segmentation algorithm could not reliably identify the FI or showed inconsistent segmentation. There were 10 trials in which the bounding box centroid was chosen as the aimpoint over the segmentation model aimpoint during testing, with an average time to aimpoint identification of 52.5 s (SD = 33.4). The majority of these cases occurred when the fascia was very shallow, so the target was near the edge of the image and the model would not segment well with some landmark features out of view.

### 3.4. Preliminary Sciatic Nerve Detection

**Figure 9:**
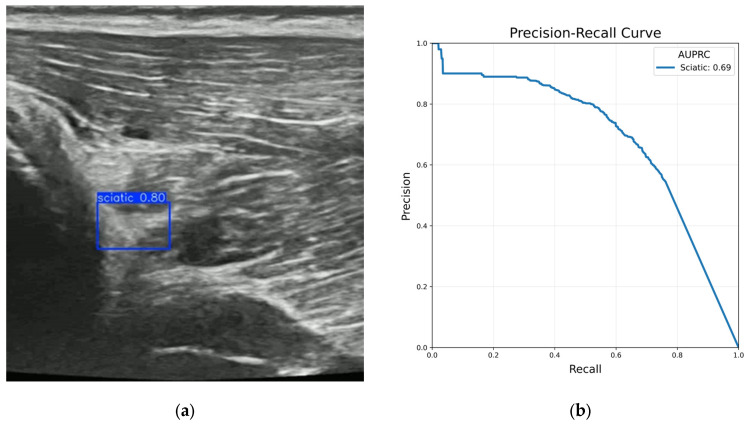
(**a**) Example ultrasound image of the sciatic nerve with a bounding box detection. (**b**) Precision–recall curve of the sciatic nerve detection model on in vivo test data with an AUPRC of 0.69.

While the primary aim of AI model development was for femoral nerve block, we separately investigated AI models for sciatic nerve (SN) detection using a small amount of in vivo porcine data. A sciatic nerve block involves needle insertion and analgesia injection into the gluteal, subgluteal, or popliteal area around the SN [34]. Figure 9 displays an example ultrasound image with the SN detected. The average four-fold cross-validation AUPRC of the SN detection model was 0.69, also shown in Figure 9. The lower performance of the SN model is related to a number of factors, including the inherent difficulty identifying the SN, label noise, and limited training data with the SN in view. In humans, it is noted that it is a more difficult block to achieve, due to its depth and lack of nearby structures, whereas the femoral block is near the more obvious FA and FV landmarks [34]. Porcine structures are generally similar to human structures in this area [35]. Although the SN detection model exhibited less robust performance, it shows that the algorithm approach can be adapted for multiple nerve block sites.

## 4. Discussion

This study aimed to address the initial challenges of performing conventional and ultrasound-assisted nerve blocks in critical care settings and orthopedic surgery by automating ultrasound image interpretation for needle aimpoint selection. The current paradigm of extensive medical training for peripheral nerve blocks often limits this pain reduction option to only larger medical facilities with robust staffing [10]. AI-enabled robotic technology has the potential to reduce the expertise needed to perform a full nerve block workflow by integrating image interpretation, nerve localization, aimpoint guidance, and needle insertion to deliver local anesthesia.

Our AI algorithms show promising results for femoral landmark detection and segmentation in porcine experiments. In comparison to FA and FV identification, the FI and nerves are more challenging to identify, have a wider variety of shapes, and can deform during ultrasound compression. Nonetheless, our models achieved average AUPRC of 0.92 for all classes on in vivo data and Dice similarity coefficients above 0.90 in 91% of segmentations. Our initial training on the SN also indicates that the AI framework could be transferred to other nerve structures successfully, given sufficient data. In future work, we aim to correct failure modes observed in the AI algorithms, particularly the reduced segmentation precision of the FI when it is highly compressed, partially occluded near the boundary of the image, or distorted by needle insertion or anesthetic injection. Deformable convolution networks could be explored to handle complex shape variations of the FI. Future work could also investigate techniques to track the position of each class in prior frames (e.g., optical flow or TAPIR [36]) and reduce false positives that correspond to large shifts in position. In practice, a single frame with a false positive does not impact the operator guidance substantially, since detections are occurring at 30 frames per second and occasional misses are overcome by the high average precision and recall.

AI guidance for peripheral nerve blocks has been explored previously to train clinicians to identify nerves [37]. These technologies help train non-specialists more quickly, but they do not cover the full workflow required to complete a peripheral nerve block, as they do not include steps for needle targeting or needle insertion [38]. The solutions on the market for ultrasound-guided nerve blocks with AI guidance are limited, and they are intended for use in hospitals and other well-resourced clinical situations [13,38,39].

When faced with a critical pain management situation in a prehospital setting or on the battlefield, responders with limited training need to quickly identify nerve locations and determine the correct path to insert a needle. Our results for time-to-aimpoint identification in porcine testing support this requirement, allowing the user to identify the neurovascular landmarks and align the device over a hypothetical aimpoint in just 40 s on average. As a comparison, one study found that medical residents, a less experienced but highly trained population, completed ultrasound-guided blocks with a median time of 1.8 min (108 s) using traditional ultrasound methods, including time to inject the anesthetic [40]. While our AI algorithms need additional development to ensure safe and accurate needle insertion and anesthetic delivery, the ability to identify structures and target a specific area in less than a minute establishes a benchmark for future studies and for users who are less familiar with nerve blocks.

We have trained the AI system to identify the femoral and sciatic nerve regional landmarks. Despite strong performance overall, there are opportunities for improvement. Our prior experience in the vascular access domain has shown that algorithm performance can be enhanced by iteratively retraining with more labeled data. Similarly, as our nerve block algorithms are trained with more nerve-region data, their generalizable performance is expected to improve compared to initial versions. Future work will also explore more aggressive image augmentation settings like random cropping, resizing, scaling, and shearing to mitigate failure modes in FI segmentation. Additionally, we aim to develop diffusion/flow matching generative image models to create synthetic images with a distorted FI for use in training the segmentation model. As the segmentation algorithm performance improves, there will no longer be a need to use the bounding box for calculating needle aimpoint, thereby increasing robustness. Practically, a compressed FI is not an ideal scenario for needle insertion, so if the algorithm is not segmenting it well, that would prompt the operator to move to a better location for a nerve block.

We plan to gather data and apply the algorithm framework to additional nerve structures, including further developing sciatic nerve identification, as well as working with complex nerve structures like the brachial plexus. These new sites have different anatomical landmarks and require unique analgesia delivery workflows, which can create new challenges due to different obstacles for the needle or obscured views. There will be additional data collection required to properly train on these structures, as well as experimentation on the best location or locations for a nerve block, as sometimes multiple injections are required. These areas will pose new areas of research and AI model development. Human data can also be gathered for algorithm training, and non-invasive human testing can then be conducted. For both animal and human validation and testing, developing algorithms on diverse populations of data, with varying size, shapes, and BMI, will improve the generalizable performance. We aim to include more ex vivo and hypotensive data into the training set to improve model generalization in future studies. In prolonged field care, the intent is to perform a nerve block in the presence of perfused tissue, not necrotic tissue, so the ex vivo data does not fully represent this scenario. Diverse data collection would also enhance the safety of AI-guided nerve blocks and increase likelihood of successful pain relief outcomes that do not require additional local or general anesthesia [13].

A limitation of the work reported in this paper is that we did not evaluate the complete nerve block procedure with needle insertion and analgesia delivery. Future development will include aimpoint refinement and integrated testing of the AI algorithms with the robotic subsystem for needle insertion in porcine models. Additional improvements on AI-GUIDE Nerve Block will address several issues across technical development, validation and testing, and applications. For example, it can be difficult to determine precisely where the needle should be placed for an effective block. The needle needs to be placed within the FI but should not contact the femoral nerve, which lies in the lower medial portion of the FI and can be difficult to visualize until fluid is injected around it. Due to variations in the angle of needle insertions, the needle, particularly the tip, can disappear from the ultrasound field of view [13]. Tracking the tip can also be difficult if it enters a hyperechoic region like the FI [13]. Mitigating these factors through needle tracking algorithms would improve not only the efficacy of the nerve block but also ensure the block is done safely. Further, to prevent intravascular injection, an automated blood flashback detection capability could be adapted from our prior work on vascular access, which employed optical sensors. The pathway to clinical translation requires improving the human factors of the device operation, guiding an untrained operator through all steps of the nerve block procedure semi-automatically, and preventing unintended complications or failure modes, such as perforating the nerve or a vessel, injecting too deep or shallow, or placing the device on the wrong part of the body. The guidance system would need to be refined through a rigorous human factors and usability analysis to ensure it addresses the realistic challenges of performing nerve blocks in clinical or battlefield medicine scenarios. We recognized that identifying the needle aimpoint is only one piece of the overall device workflow and clinical translation challenge.

The AI-GUIDE Nerve Block technology has the potential to enable better pain management in prehospital environments, underserved communities, and hospitals with gaps in personnel expertise. Making ultrasound-assisted blocks available to medical staff without extensive training would improve the economics and efficacy of pain management and help to curb opioid usage for pain [1,2,3]. As a highly portable device, AI-GUIDE Nerve Block could be used in medical transport, on the battlefield, or even during space travel, as peripheral blocks sidestep problems with airway management and other challenging conditions in microgravity [41,42]. Developing countries would also benefit from having access to this relatively safe and inexpensive procedure, which so far has been limited by training opportunities and a slow adoption of regional nerve blocks [43].

## 5. Conclusions

In summary, this work presented the development and testing of deep learning-based algorithms to interpret ultrasound images, detect nerve region landmarks, and calculate an aimpoint for needle insertion. The prototype focused on automating the initial steps in the femoral nerve block procedure. Prospective porcine testing of the technology demonstrated fast and effective needle aimpoint identification by users with limited medical expertise. The AI algorithms were evaluated under both normovolemic and hypovolemic conditions, showing robust performance in hypothetical emergency scenarios involving hemorrhagic shock. Future work will focus not only on improving algorithm performance but also integrating with hardware for needle insertion and analgesia delivery. By combining AI, ultrasound, and robotic needle placement into a single handheld device, the AI-GUIDE Nerve Block would empower more personnel to perform peripheral nerve blocks efficiently, improving procedural safety, accessibility, and patient outcomes in both mainstream hospitals and resource-limited environments. The results on algorithm development presented in this study mark a significant first step towards realizing that potential.

## 6. Patents

MIT Lincoln Laboratory and United States Army Institute of Surgical Research have jointly filed a provisional patent application with the United States Patent and Trademark Office (USPTO) related to the work reported in this manuscript. The provisional application was filed on 14 April 2025 and is titled “Systems and methods for artificial intelligence and ultrasound guided intervention” (Application No. 63/788,402). The inventors listed on the application include L.J.B., B.A.T., L.A.G, J.W., S.B.K., B.J.K., E.J.S., C.B., and A.C. This patent covers the core methodology presented in this study for AI-enabled, ultrasound-guided needle-based procedures including nerve blocks.

## Figures and Tables

**Table 1:** Summary of datasets used for algorithm development.

	Femoral Nerve		Sciatic Nerve
AI Model Type	Detection	Segmentation	Detection
Ultrasound Images	55,455	53,877	13,668
Number of Swine	20 (17 in vivo and 3 ex vivo)		4 (in vivo)

**Table 2:** Tested hyperparameters used for the five-fold cross-validation training of the femoral landmark detection model, with final hyperparameters in bold.

Batch Size	Learning Rate (Max)	Optimizer	Weight Decay	Image Size
**16**, 64, 256	1 × 10^−2^, 1 × 10^−3^, **1 × 10^−4^**	Adam, **AdamW**, SGD	**1 × 10^−3^**, 1 × 10^−4^	**320**, 640

**Table 3:** Summary of attempt success rates and timing for the image processing algorithms to guide the operator to a suitable needle insertion aimpoint in the ex vivo models.

Parameter	Round 1	Round 2	Round 3	Round 4
Number of Ex Vivo Porcine Models	3	3	3	2
Number of Operators	5	6	2	2
Attempts	9	38	67	46
Successful Aimpoint Identifications	2	28	65	43
Success Rate	22.2%	73.7%	97.0%	93.5%
Mean Time per Attempt (s)	433.3	193.2	47.7	96.1
Standard Deviation Time per Attempt (s)	158.1	167.1	46.2	100.4

## Data Availability

The datasets presented in this article are not publicly available because they have been collected and maintained in a U.S. government-controlled database that is located at MIT Lincoln Laboratory and the U.S. Army Institute of Surgical Research. Data may be made available through the development of a collaborative agreement with the corresponding author. The underlying code for this study and training/validation datasets may be made available to qualified researchers on reasonable request from the corresponding author pending U.S. Government approval.

## Supplementary Materials

The following supporting information can be downloaded at: https://www.mdpi.com/article/10.3390/bioengineering13050556/s1, Video S1: AI-GUIDE Nerve Block prospective live porcine testing.

## Abbreviations

The following abbreviations are used in this manuscript:

AUPRC	Area under the precision-recall curve
FA	Femoral artery
FI	Fascia iliaca
FV	Femoral vein
SD	Standard deviation
SDK	Software development kit
SN	Sciatic nerve
